# Characteristics of abdominal herniation and its associations among patients operated in a Sudanese tertiary hospital: a retrospective review

**DOI:** 10.1186/s12893-024-02741-4

**Published:** 2025-01-04

**Authors:** Ahmed Abdellateef Hassan Bakry, Khabab Abbasher Hussien Mohamed Ahmed, Mohammed Mahmoud Fadelallah Eljack, Ghassan E. Mustafa Ahmed

**Affiliations:** 1 Faculty of Medicine, University of Khartoum, Khartoum, 11111 Sudan; 2 Faculty of Medicine, University of Bakht Alruda, Al Dwuaym, Sudan

**Keywords:** Abdominal wall, Surgery, Hernia, Anatomy

## Abstract

**Background & aims:**

Hernia is a very common surgical condition affecting all ages and both sexes. Data regarding abdominal wall hernias is essential to hernia management in an institution. With the absence of data regarding the prevalence, characteristics, and associations of abdominal wall hernias in Sudanese patients, we aimed to describe and find the possible differences in the spectrum of abdominal hernias, their rates, and associated predisposing factors.

**Methodology:**

This was a retrospective cross-sectional chart review of surgical patients admitted at a Sudanese tertiary teaching Hospital, Department of Surgery, from January 2019-December 2021. Data were collected from the medical records using a checklist. The data obtained included age, gender, occupation, chronic medical conditions, past medical history (PMH), and year of admission. Data were analyzed using SPSS 22.

**Results:**

Results showed that between January 2019 and December 2021, a total of 1158 patients were admitted to the department of surgery, and abdominal hernia had a frequency of 16.23% (*n* = 188). There was a male predominance (55.3%), ages below 20 years constituted the majority of cases (45.2%), and those between (50–60 years) were the least. The most frequent subtype was the inguinal hernia. The association between sociodemographic variables, PMH, chronic medical conditions, and the diagnosis was found to be statistically significant. The rate of recurrence was found to be 10.1%.

**Conclusion:**

There was a high rate of abdominal Herniation, and a difference between subtypes of abdominal herniation regarding demographic data, past medical history, and comorbidities.

**Supplementary Information:**

The online version contains supplementary material available at 10.1186/s12893-024-02741-4.

## Introduction

A hernia is characterized by an abnormal bulging of a viscus through a defect in the wall of the cavity containing it. This can manifest through the anterior abdominal wall (ventral) as umbilical/paraumbilical, epigastric, or, less commonly, Spigelian hernia, or within the groin as an inguinal hernia or femoral hernia [1]. Wall defects may arise from congenital anomalies, such as umbilical and diaphragmatic hernias, or be acquired through trauma or abdominal surgery [2]. A normal abdominal wall typically withstands high abdominal pressure, preventing herniation. However, hernias have been associated with conditions like constipation-induced high pressure, certain prostatic symptoms, excessive coughing from respiratory diseases, and obesity [2]. Interestingly, hernia occurrence is not higher among Olympic weightlifters compared to the general population, suggesting that high pressure alone is not a significant factor in hernia development [3]. Many individuals notice a hernia after severe straining. Moreover, strong evidence indicates that collagen abnormalities are scientifically established etiological factors for abdominal wall hernias, supported by histological evidence and links to other collagen-related diseases such as aortic aneurysm [2].

A research study carried out in Addis Ababa, Ethiopia, revealed that incisional hernia is the predominant type of abdominal hernia, representing 52.3% of all cases, followed by inguinal hernias at 33.8% [4]. Previous research has identified various factors strongly associated with external hernias, including muscular weakness, multiple pregnancies, surgical history, gender, age, chronic cough, constipation, smoking, physically demanding work, and a family history of hernias [5]. Hernias rank among the most prevalent surgical issues, contributing to considerable morbidity and mortality across different regions in Africa [6]. If left untreated, hernias can result in severe complications, such as strangulation, incarceration, and intestinal obstruction. Strangulation, in particular, is an acute surgical emergency that can have dire outcomes [7]. In Nigeria and Sudan, strangulated external hernias were the leading cause of intestinal obstruction, accounting for 56.9% and 27.7% of cases, respectively [8].

Abdominal hernia repair is among the most common surgical procedures, with a prevalence ranging from 100 to 500 per 100,000 people, typically performed electively [2]. The primary complication of abdominal wall hernias is the potential incarceration, obstruction, entrapment and strangulation of intestinal parts, leading to a surgical emergency with high morbidity and mortality. In anterior abdominal wall hernia cases, emergency surgery due to incarceration is required in 5–13% of cases, with 10–15% requiring intestinal resection and anastomosis due to intestinal ischemia [9, 10]. Incarcerated anterior abdominal wall hernias can occur at inguinal, femoral, umbilical, and incision sites, with patients commonly presenting to the emergency department with localized swelling and pain, posing life-threatening risks from strangulation and obstruction of the trapped organ [11]. Mortality and morbidity rates are elevated in elderly patients with associated comorbidities and when interventions are delayed beyond 24 h [3, 12, 13]. Thus, we advocate for elective surgery for abdominal wall hernias, even in asymptomatic cases, to prevent complications in older age [14].

Successful repair of groin hernias depends on adequate knowledge of groin anatomy and physiology. Hernias can be repaired using open tissue or mesh, as well as minimally invasive procedures, with surgeons typically choosing the method they are most familiar with or aligning with guidelines for ensuring best outcome [15]. The use of mesh in hernia repair depends on the defect’s size, muscle weakness, and the surgeon’s preference. Theoretically, reducing the therapeutic density of polypropylene to create lightweight mesh lessens the foreign body response, enhances abdominal wall compliance, decreases mesh contraction or shrinkage, and facilitates better tissue incorporation [15]. There is a growing trend towards synthetic mesh repair in various hernia settings. The use of mesh in adult umbilical and incisional hernias has increased from 32% and 34.6–63.8% and 90.7%, respectively [16]. Ventral hernia repairs range from anatomical repair and darning to open mesh repair [16]. The increasing popularity of synthetic mesh in hernia surgery suggests growing confidence in this repair method [16]. Laparoscopic repair is considered superior to open mesh repair due to significantly less blood loss, fewer complications, shorter hospital stays, and better cosmetic outcomes, though it has disadvantages like longer operation times, higher costs, and a steeper learning curve [17].

Given the lack of data on the prevalence, characteristics, and associations of abdominal wall hernias in Sudanese patients, our study aimed to estimate the proportion of abdominal hernias, characterize different subtypes, and explore associations between diagnoses and various predisposing factors, including past medical history and chronic medical conditions, from January 2019 to the end of December 2021.

## Methodology

This was a retrospective cross-sectional chart review which was carried out at the surgical department of a Sudanese tertiary teaching hospital, in Gezira state, Sudan from January 2019 till the end of December 2021. The surgical ward of the hospital is composed of 20 beds. About 20 operations are done per week, while the surgical department contains two surgical units containing two specialists and 4 house officers. A retrospective review of records of surgical patients was carried out. Total coverage of all the medical records of patients admitted at the department of surgery in the hospital 2019–2021 was adopted. Data was collected using a checklist composed of the following variables: Socio-demographic information: including age, gender, marital status, and occupation, year of admission: 2019.2020.2021, chronic medical conditions (the open-ended question was designed to investigate the presence of chronic disorders), past medical history i.e. history of previous hospitalization or surgical procedures, etc.) (the open-ended question was designed to investigate the past medical history of cases). The questionnaire was developed for this study and validated via a pilot study (see the Supplementary File). The collected data were entered into Microsoft Excel and results were generated using SPSS version 22. Mean, range, median, and frequencies were used for descriptive data. Pearson chi-square test was used to study the associations of Diagnosis with socio-demographic characteristics, past medical history, chronic medical conditions, and year of admission. This work has been reported in line with the STROCSS criteria [18].

## Results

**Fig. 1:**
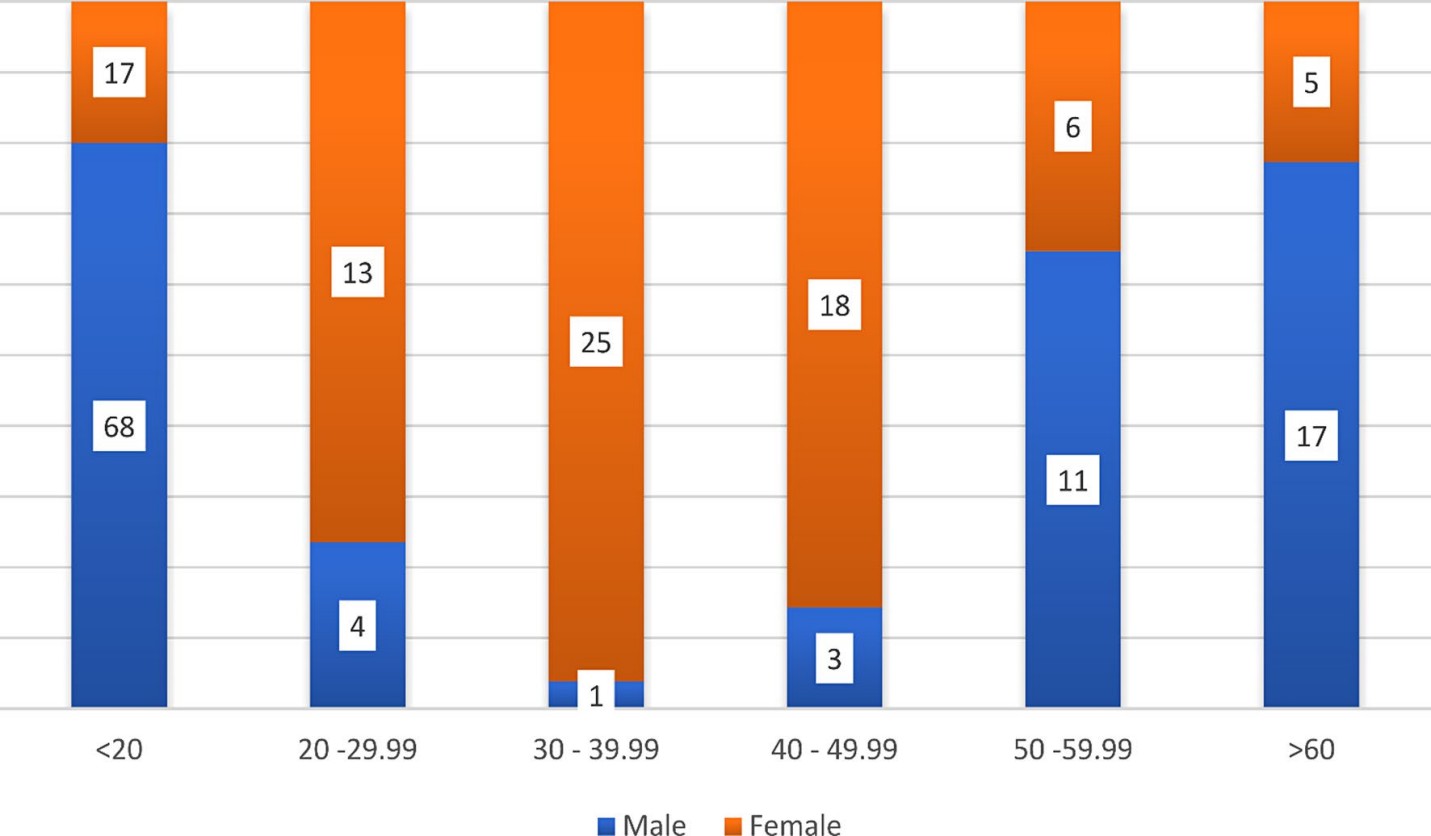
Distribution of age groups among the patients in the study

The mean age of the study group was 27.07 years, and the age range of the sample was 84.33 years, ranging from 8 months − 85 years (see Fig. 1).

Between January 2019 and December 2021, a total of 1158 patients were admitted to the department of surgery, with a frequency of abdominal hernia of 16.23% (*n* = 188).

Ages below 20 years constituted the majority of cases 45.2, followed by groups 3, 6, 4, 2 and 5 shared the last place.

Males constituted the majority of the total cases (55.3%). The most frequent cases were in group 1 and group 6 respectively, while in females the most frequent cases were in group 3, followed by group 4 and group 6 was the least to develop hernia.

### Marital status

See Fig. 2.

**Fig. 2:**
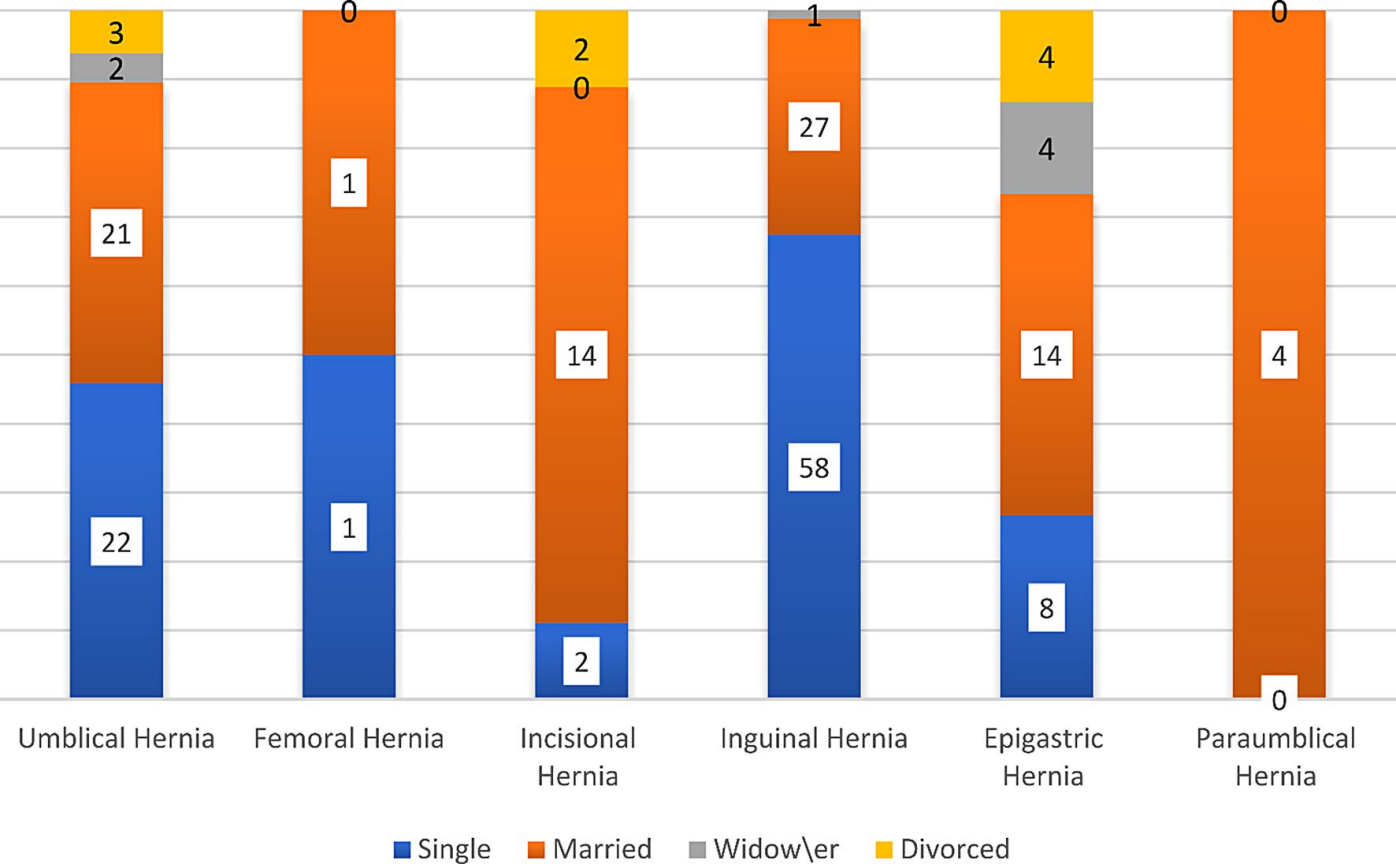
Differences of marital status among patients diagnosed with hernia subtypes

### Occupation

The most common occupation found among patients was “student”, which constituted 25.5% of cases, followed by housewife 24.5% and the least occupation documented was “nurse” 1.1%.

**Fig. 3:**
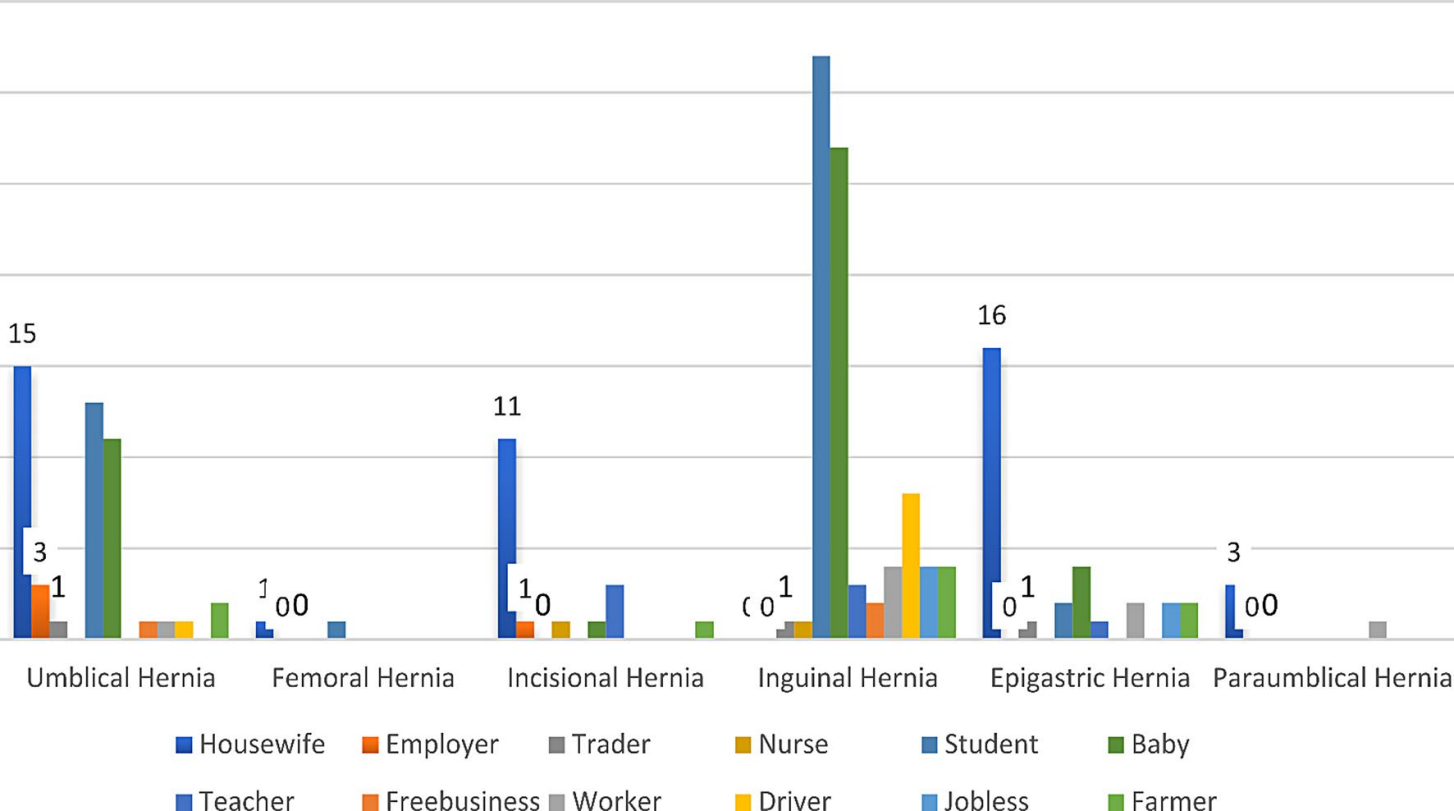
Occupations of patients in contrast to each subtype of hernia

The most common occupation to be documented among males rather than being a “child” which constituted about 33.65% of the male gender was “student”. While among females, the most common occupation was “Housewife” which constituted 54.76% of the female gender (see Table 1; Fig. 3).

**Table 1:** Lists the descriptive statistics of Occupation among the patients in the study

Occupation	Frequency	Percent
Housewife	46	24.5
Employer	4	2.1
Trader	3	1.6
Nurse	2	1.1
Student	48	25.5
Child	43	22.9
Teacher	7	3.7
Free business	3	1.6
Worker	8	4.3
Driver	9	4.8
Jobless	6	3.2
Farmer	9	4.8
Total	188	100.0

### Distribution of cases by year

Cases increased annually, by 2019 it was 30.3, in 2020 it was 30.9 and in 2021 it was 38.8. In all the past 3 years, inguinal hernia constituted the most abundant type. In 2019, femoral and paraumbilical hernias were the least while in 2020, there was no femoral hernia and 75% of cases of paraumbilical hernias occurred in 2020. In 2021, there was no paraumbilical hernia and the least hernia to occur was a femoral hernia.

### Diagnosis and predisposing factors

Inguinal hernias were the most common at 45.7%, followed by umbilical hernia at 25.5%, epigastric hernia at 16.0, incisional hernia at 9.6, paraumbilical at 2.1, and femoral hernia 1.1 (see Table 1).

About 41.7% of cases of hernia in females were umbilical hernia, which was the most common abdominal hernia, followed by epigastric hernia 26.2 and incisional hernia 20.2.

Inguinal hernia constituted the most common type of hernia cases in males 78.8%, followed by umbilical hernia12.5% and epigastric hernia 7.7% (see Table 2).

**Table 2:** Lists the descriptive statistics of hernia subtypes among the patients in the study

Diagnosis	Frequency	Percent
Umbilical hernia	48	25.5
Femoral hernia	2	1.1
Incisional hernia	18	9.6
Inguinal hernia	86	45.7
Epigastric hernia	30	16.0
Paraumbilical hernia	4	2.1
Total	188	100.0

Regarding the relation between gender and diagnosis, males were predominant in most cases in all types of hernia except inguinal hernia.

Among males, patients below 20 years were the majority of cases followed by those above 60 years, while in females the majority of cases were 30–39 years and patients above 60 years were the fewest with only 5 cases out of 84 cases (see Table 3).

**Table 3:** Lists the relation between Hernia subtypes and different variables among patients in the study

	Diagnosis							
	Umbilical hernia	Femoral hernia	Incisional hernia	Inguinal hernia	Epigastric	Paraumbilical	Total	Pearson chi-square
***Gender***								
Male	35	2	17	4	22	4	84	0.000
Female	13	0	1	82	8	0	104	
Total	48	2	18	86	30	4	188	
***Marital status***								
Single	*22*	1	2	58	8	0	91	0.000
Married	21	1	14	27	14	4	81	
Widow	2	0	0	1	4	0	7	
Divorced	3	0	2	0	4	0	9	
Total	48	2	18	86	30	4	188	
***Occupation***								
House wife	15	1	11	0	16	3	46	0.000
Employer	3	0	1	0	0	0	4	
Trader	1	0	0	1	1	0	3	
Nurse	0	0	1	1	0	0	2	
Student	13	1	0	32	2	0	48	
Baby	11	0	1	27	4	0	43	
Teacher	0	0	3	3	1	0	7	
Free business	1	0	0	2	0	0	3	
Worker	1	0	0	4	2	1	8	
Driver	1	0	0	8	0	0	9	
Jobless	0	0	0	4	2	0	6	
Farmer	2	0	1	4	2	0	9	
Total	48	2	18	86	30	4	188	
***Age***								
< 20.000	21	1	1	57	5	0	85	0.000
20.000–29.999	7	0	2	3	5	0	17	
30.000–39.999	8	0	9	0	8	1	26	
40.000–49.999	9	1	4	2	4	1	21	
50.000–59.999	3	0	0	9	4	1	17	
60.000+	0	0	2	15	4	1	22	
Total	48	2	18	86	30	4	188	
***Year of Admission***								
2019	16	1	5	27	7	1	57	0.176
2020	12	0	7	31	5	3	58	
2021	20	1	6	28	18	0	73	
**Total**	**48**	**2**	**18**	**86**	**30**	**4**	**188**	

In patients with a history of vaginal delivery, the most common hernia to develop was umbilical hernia followed by epigastric and inguinal hernia.

The most common subtype to recur was an incisional hernia, while those with femoral hernia and the paraumbilical hernia were the least to present with recurrent hernia.

In patients with a history of surgical operation the most documented subtype of abdominal herniation was incisional hernia followed by inguinal hernia, umbilical, epigastric, paraumbilical and femoral hernias respectively.

In patients with a history of caesarean section, the most common hernia to complicate was incisional hernia followed by epigastric and paraumbilical/umbilical. This finding was statistically significant (p-value < 0.05).

History of similar condition was in 19 patients with incisional hernia being the most common hernia to recur followed by inguinal, epigastric, umbilical and paraumbilical respectively. The femoral hernia wasn’t documented to recur in our data (see Table 4).

**Table 4:** Lists the relation between Hernia subtypes and different variables among the patients in the study

	Diagnosis							
	Umbilical hernia	Femoral hernia	Incisional hernia	Inguinal hernia	Epigastric	Paraumbilical	Total	Pearson chi-square
***Past Medical History***								
Vaginal Delivery	8	0	0	1	2	0	11	0.010
Similar Condition	3	0	7	4	4	1	19	0.001
Surgical Operation	2	0	17	5	2	1	27	0.000
Hospitalization	0	0	0	1	0	0	1	0.946
Caesarean Section	1	0	8	0	3	1	13	0.000
Blood Transfusion	0	0	0	0	0	1	1	0.000
***Comorbid Conditions***								
Diabetes Mellitus	3	0	1	2	2	0	8	0.846
Asthma	1	0	0	3	2	0	6	0.824
Hypertension	*2*	0	5	3	1	1	12	0.003
Anemia	0	0	0	2	0	0	2	0.792
Hyperacusis	0	0	0	0	1	0	1	0.381
Chronic Cough	18	2	7	15	9	1	52	0.021
Hypothyroidism	1	0	0	0	0	0	1	0.710
Nephrotic Syndrome	1	0	0	0	0	0	1	0.710
Benign Prostatic Hyperplasia	0	0	0	1	0	0	1	0.946

## Discussion

This is the first study ever, up to our knowledge and the hospital administration and staff to be conducted in a Sudanese tertiary hospital regarding characteristics of abdominal herniations and their association. This study aims to assess the characteristics of abdominal hernia and its associations among patients in the study area. A total of 1158 patients were included in the department of surgery, with an abdominal hernia frequency of 16.23% (*n* = 188).

(2019, femoral and paraumbilical hernia were the least. In 2020, there was no femoral hernia, and in 2021, there was no paraumbilical hernia and the least hernia to occur was femoral hernia. Such variability between the inguinal and femoral hernia is attributed to the nature of the inguinal hernia which commonly occurs due to occupational, or pressure factors encountered daily. In contrast, femoral hernia is mostly associated with females who are either obese or pregnant.

Regarding the association between age and diagnosis for the total cases - which was found to be statistically significant (*P* = 0.000) - ages below 20 years constituted the majority of cases (45.2%), while those between [20–30] and (50–60) had the least rate to develop abdominal hernias. This is justifiable since individuals below 20 are more active and involved in strenuous occupations or sports, rendering them vulnerable to increased intra-abdominal pressure and subsequent herniation. The majority of cases in patients under 20 years were inguinal and umbilical hernias and the cases especially among children are most likely to be explained as congenital conditions.

When describing our population’s gender, our study found that males (55.3%) were more common than females (44.7%), but despite those females constituted the majority of cases in all types of hernia except in inguinal hernia and this relation between gender and diagnosis was statistically significant. The most common subtype of herniation among females was umbilical hernia, followed by epigastric hernia and incisional hernia, which was statistically significant in patients with a history of vaginal delivery and caesarean section. The most common subtypes to occur among males were inguinal hernias and the least hernia to be documented rather than femoral and paraumbilical was incisional hernia.

In our study, inguinal hernias were the most common (45.7%) followed by umbilical hernia (25.5%) as the 2nd most common subtype. However, a previous study [1] which was done to describe and find the possible differences in the spectrum of abdominal hernias and document trends in their management concluded that inguinal hernias were the most common (56%), and femoral hernias were the least (0.87%). Other studies also mentioned that inguinal hernia was the most common hernia. (73% of cases) [20, 21]. Various studies agree that inguinal (70–75%), femoral (6–17%), and umbilical (3–8.5%). The most prevalent of these hernias is inguinal hernia, which was also listed in Maingot’s abdominal operations [22].

To mention the relation between past medical history and diagnosis, our study found a result that described having a medical history of vaginal delivery, similar condition, surgical operation, caesarean section and blood transfusion to be statistically significant by *P* = 0.010, 0.001, 0.000, 0.000, and 0.000 respectively. This can be explained by the fact that there is a significant risk of recurrence among patients presented at our hospital, surgical operations and improper suturing can all contribute to the development of abdominal herniation.

In patients with a history of vaginal delivery (*P* = 0.010), the most common hernia to develop was umbilical hernia followed by epigastric and inguinal hernia and according to an Egyptian study done by Dr. Samir Ahmad Ammar and his colleague who came to a result that early marriage and repeated pregnancy may be partially responsible for this relatively high percentage of adult umbilical hernias [16].

There were 19 surgeries (10.1%) for patients with a history of similar condition (*P* = 0.0010), among these patients the most common subtype was recurrent incisional hernia which was 3.7% of the total cases of abdominal herniation.

In patients with a history of surgical operation (*P* = 0.000), the most documented subtype of abdominal herniation was incisional hernia (9.04%), and the least was a femoral hernia. In a similar study one of its objectives was to describe the clinical profile of anterior abdominal wall hernias found that The incisional hernia rate was 9% found by Dr. O.O. Ayandipo and his colleagues and their results – like ours - was closer to the rates described in the western world of 6–10% [1], but higher than rates (1–4%) quoted by various authors in Africa [23, 24, 6].

### Inguinal hernia

Inguinal hernia repair consumes a lot of healthcare resources because it has a high lifetime risk; 27% for men and 3% for women [25].

Inguinal hernias are known to be the most common subtype of hernia as mentioned above. Our study found that the ratio of inguinal hernias to other types was 1:1.2, while another study done in Ibadan, Nigeria study said the ratio of inguinal hernias to other types was 3:1 [23]. The results of another study show an inguinal to other hernia ratio of 3:1 (75.9%) which is at par with the 75% quoted by various other authors [25–27]. In contrast, a similar study in Ethiopia reported that the epigastric hernia had the highest prevalence (34%), followed by inguinal hernia (29.8%) [28]. This difference could be attributed to methodological variations in terms of sample size and sample selection.

In this study, males showed a predominance regarding having inguinal hernias compared to females, by a ratio of 20.5: 1, other authors came to a conclusion closer to ours illustrating that males are said to be 20 times more likely than females to have inguinal hernias [29]. Another study was conducted by Emeka Ray-Offor who found that the male-to-female ratio was 7:1, however femoral hernia had was predominant among females [1]. In our study, 95% of inguinal hernias were operated on in males, which is close to an Egyptian study which found that 96% of inguinal hernia operations were performed on males [16]. The most common occupations to develop inguinal hernia were “student” and “child”, so the most frequent age group was found to be group 1 (< 20 years), and that can be explained by the high risk of developing congenital inguinal hernia among children.

### Umbilical hernia

About 25% of the total cases of abdominal herniation occurred in patients above 20 years and it was the most frequent hernia subtype among females as mentioned. Textbooks also quote the rate of umbilical/para-umbilical hernia to be up to five times commoner in women, citing pregnancy as a significant etiological factor [29, 30].

The most frequently documented occupation to develop this subtype of hernia was “Housewife” and most of them were single (*n* = 22), the high rate of umbilical hernia among this population can be explained by that obesity – which wasn’t documented in the medical records -physical exertion and pregnancy could have facilitated in the development of umbilical herniation among these occupations [19].

### Epigastric hernia

Regarding epigastric hernia (16.0%), In our study 73.3 of total cases of epigastric herniation were operated on in females while 26.666 were in males, In an Egyptian study, Epigastric hernias were twice more common among males than females (67.5% versus 32.5% respectively) [16]. The most documented age group to develop epigastric hernias is those in group 3 (30–40) years.

The most common occupation found among those patients was “housewife” (24.5%) and the majority of them were single (48.4%). This can be explained by the fact that Obesity, physical exertion, and pregnancy are thus key etiological variables in the development of epigastric hernias and these were illustrated in a British study which was conducted over a 3 times period, where in England, the prevalence of obesity among males increased from 13.2% in 1993 to 23.1% in 2005 and from 16.4 to 24.8% for females during the same period [19].

### Incisional hernia

The incisional hernia has a proportion of (9.6%), while In an Egyptian study, incisional hernia constituted (10.3%) [16], The incisional hernia rate was 9% found by Dr O.O. Ayandipo and his colleagues and their results – like ours - were closer to the rates described in the Western world of 6–10% [2], but higher than rates (1–4%) quoted by various authors in Africa [23, 24]. The most occupation documented among patients diagnosed as having incisional hernia was “housewife”.

### Paraumbilical hernia

Regarding paraumbilical hernia, we found a frequency of (2.1%) which was closer to Other studies that mentioned that it was one of the rarest types to be found (1–2%) [18, 19]. On the other hand, an Egyptian study found a frequency of (22.7%) [16].

The most common occupation found among those patients was “ housewife” and the majority of them were married this can be explained by the fact that Obesity, physical exertion, and pregnancy are thus key etiological variables in the development of paraumbilical hernias and these were illustrated in a British study which was conducted over a 3 times period [19].

### Femoral hernia

Femoral hernia is known to be the third most prevalent kind of primary hernia, according to traditional textbooks [29, 30]. In our study, femoral hernia has a rate of (1.1%) and a female predominance. In a British study, the rate of femoral hernia was only 3.7% equating to the fifth commonest hernia type [19].

### Strength of the study

According to our knowledge, a limited number of Sudanese studies were published in this study areas; hence this study is considered a valuable base for evidence and a significant addition to the database. Furthermore, this study included participants from multiple demographic backgrounds and socio-economic statuses, which would aid the authorities in managing the issue from all aspects.

### Limitations of the study

One limitation of this study is the narrow study area, which may have determined a highly selected group of respondents. Hence, the generalizability of the findings to the total community of patients may be difficult. This can limit the applicability of the study’s conclusions in different settings or demographics. Bigger numbers of respondents would have improved the statistical significance of the results. Another limitation of this study was its retrospective design and the above incompetency in filling the files. This can lead to missing data or misinterpretation of the information, affecting the validity of the findings.

## Conclusion

This study described the characteristics of patients with abdominal herniation and its associations admitted at a tertiary Sudanese teaching hospital, specifically at the department of surgery for three consecutive years. The study shed the light on a potential association between the variable characteristics of patients (such as physical exertion and BMI) and the specific subtype that they were admitted for in the hospital. The male patients in this study were a majority, and the most common abdominal hernia subtype was inguinal hernia, with a relatively high rate of recurrence.

### Recommendations

- Further studies need to be done to look for other predisposing factors for hernia in this area.
- An audit program needs to be established to promote the overall records filling in the hospital and to make documentation more appreciated to reach proficiency.
- There must be an electronic system to keep the patient’s data more secure and easily reachable for research or medical purposes.

## Electronic supplementary material

Below is the link to the electronic supplementary material.

Supplementary Material 1

## Data Availability

The data that supports the findings of this study is available with the corresponding author upon reasonable request.
