# Supplementary material for: Characteristics of abdominal herniation and its associations among patients operated in a Sudanese tertiary hospital: a retrospective review
Source: BMC Surg. 2025 Jan 4;25:3. doi: 10.1186/s12893-024-02741-4 (PMC11699653; doi:10.1186/s12893-024-02741-4)
Supplement: Supplementary file 1 — Supplementary Material 1 [file 12893_2024_2741_MOESM1_ESM.pdf]

Data Collection Form for Retrospective Chart Review

Title

Characteristics of Abdominal Herniation and its Associations among Patients Operated in a Sudanese Tertiary Hospital: A Retrospective Chart Review

---

Instructions

This form is used to extract data from patient medical records for a retrospective study on abdominal herniation. Please ensure all data is collected accurately and completely from the charts of patients who underwent hernia surgery during the years 2019, 2020, and 2021.

Section 1: Demographic Information

1. **Age: ______________**
2. **Gender:**
   - ☐ Male
   - ☐ Female
   - ☐ Other (please specify): ___________
   - ☐ Not recorded
3. **Occupation:**
   - ☐ Employed
   - ☐ Unemployed
   - ☐ Retired
   - ☐ Student
   - ☐ Other (please specify): ___________
   - ☐ Not recorded
4. **Marital Status:**
   - ☐ Single
   - ☐ Married
   - ☐ Divorced
   - ☐ Widowed
   - ☐ Other (please specify): ___________

- ☐ Not recorded

Section 2: Medical History

5. **Comorbid Medical Conditions:** (Please check all that apply)
    - ☐ Diabetes
    - ☐ Hypertension
    - ☐ Heart Disease
    - ☐ Respiratory Disease
    - ☐ Kidney Disease
    - ☐ Liver Disease
    - ☐ Cancer
    - ☐ None
    - ☐ Other (please specify): ___________
    - ☐ Not recorded
6. **Past Medical History:** (Please list any significant past medical conditions or surgeries)
    - ______________________________
    - ______________________________
    - ☐ Not recorded

Section 3: Hernia Information

7. **Type of Hernia:**
    - ☐ Inguinal Hernia
    - ☐ Femoral Hernia
    - ☐ Umbilical Hernia
    - ☐ Epigastric Hernia
    - ☐ Incisional Hernia
    - ☐ Other (please specify): ___________
    - ☐ Not recorded
8. **Year of Surgery:**
    - ☐ 2019
    - ☐ 2020
    - ☐ 2021

Ethical Considerations

- Ensure that all extracted data is kept confidential and used only for research purposes.
- Follow institutional guidelines for accessing and handling patient medical records.
- This study has been reviewed and approved by the Ethical Board.

Data Extractor Information

1. **Name of Data Extractor:**
    - ______________________________
2. **Date of Data Extraction:**
    - ______________________________

Please ensure all sections are completed accurately. If any information is not available in the medical record, please mark it as "Not recorded."
